# Educational Intervention Improved Parental Knowledge, Attitudes, and Practices (KAP) and Adherence of Patients with Celiac Disease to Gluten-Free Diet

**DOI:** 10.1155/2020/8850594

**Published:** 2020-09-17

**Authors:** Nour Amin Elsahoryi, Eyad Altamimi, Hadil Shafee Subih, Fwziah Jammal Hammad, Jayne V. Woodside

**Affiliations:** ^1^Nutrition Department, Faculty of Pharmacy and Medical Sciences, University of Petra, P.O. Box 961343, Amman 11196, Jordan; ^2^Pediatric Department-Faculty of Medicine, Jordan University of Science and Technology, P.O. Box 3030, Irbid 21210, Jordan; ^3^Department of Nutrition and Food Technology, Faculty of Agriculture, Jordan University of Science and Technology, P.O. Box 3030, Irbid 21210, Jordan; ^4^Centre for Public Health, School of Medicine, Dentistry and Biomedical Science, Queen's University Belfast, Belfast, UK BT12 6BJ

## Abstract

**Background:**

Raising the knowledge level though education for a celiac disease patient's parents could improve the parent's adherence and practice and consequently recover the patient's adherence and symptoms and increase the patient's compliance.

**Aim:**

The present study was aimed at assessing the knowledge, attitudes, and practices (KAP) of parents who have children with celiac disease aged from 2 to 15 years old and the change in self-reported patient's adherence pre-/posteducational intervention.

**Method:**

This intervention study was designed as a quasiexperiment with evaluation pre-/post intervention analyses. Two educational sessions were carried for the parents of CD patients. A reliable and valid questionnaire was used to assess all independent variables pre-/post intervention. The parents were asked to complete the questionnaire pre and post the education sessions. The time between the sessions was two weeks.

**Results:**

100 parents were recruited, and 40 parents participated and completed the study. Baseline parent's knowledge was significantly associated with the source of information (*p* value = 0.02), while the patient's adherence was associated with the onset of disease (*p* value = 0.04). There were significant differences in the parent's KAP and patient's adherence between pre- and posteducational intervention (*p* value was ≤0.001, for all variables).

**Conclusion:**

Based on the results, this study suggested that the educational intervention increased the parent's KAP and improved the patient's adherence to the gluten-free diet significantly, which may lead to improvement in the celiac disease patients' health outcomes.

## 1. Introduction

Celiac disease (CD) is an autoimmune enteropathy triggered by dietary gluten in genetically susceptible individuals. CD is considered as one of the gluten-related disorders which is characterised by a small bowel enteropathy occurring in genetically susceptible individuals whilst exposed to the protein gliadin [1]. Gluten proteins and related prolamins found in wheat, barley, and rye trigger an autoimmune injury to the gut, skin, liver, joints, uterus, and other organs [2]. The diagnosis and the symptoms of CD are clearly reviewed in many research articles [2–6]. Briefly, the diagnosis is usually achieved through a screening blood test followed by a biopsy of the small intestine to detect villous atrophy. The symptoms vary between mild and severe among the patients, but the most common include diarrhea, poor appetite, bloated or painful belly, and weight loss or difficulty gaining weight [7, 8].

Currently, the prevalence of CD is still unknown in most countries due to the lack of awareness among the populations [6]. A recent systematic review and meta-analysis concluded that the pooled global prevalence of CD worldwide was 1.4% based on biopsy test and the condition is more common in children [6]. In the Middle East, very recent systematic reviews and meta-analyses indicate that the pooled seroprevalence is 1.6 (95% CI 1.2–2.1), and this estimate includes Iran, Turkey, Saudi Arabia, Israel, Jordan, and Egypt [6].

However, research regarding the prevalence of CD in Jordan is limited. Rawashdeh et al. found that the incidence of CD in Jordan was 1 in 2,800 live births [5]. In 2020, a serological screening study on Jordanian schoolchildren concluded that 1 : 124 (0.8%; 95% CI, 0.5% to 1.3%) was positive for CD and there was a significant difference in height and weight reduction between males and females [9]. A recent retrospective study carried out in 2017 stated that thirty-five children were diagnosed with CD in south Jordan [3]. However, Altamimi reported that the “true prevalence of the disease in our area of the world is underestimated due to the lack of awareness of the atypical presentation of the disease” [3].

The transition from a regular and strict gluten-containing diet to a GFD is considered to be the first therapeutic intervention to improve health after diagnosis [10]. This diet is a lifelong treatment, and patients and their families (especially in young patients) should be followed by a dietitian at the time of diagnosis and follow-up to provide ongoing assessment, knowledge update, and support [10]. However, little is known about the level of adherence to the GFD and how to encourage such adherence.

Providing information to the patients and the surrounding society and all sectors is a key point in the treatment. Increased awareness of primary care sectors (as physicians and dietitians) improves the detection rate, reduces patient suffering, and decreases morbidity [11]. In addition, affordable GFD appears to improve patient compliance [11]. Therefore, improving dietary adherence that defines GFD is among the main tasks of health care personnel encountering patients with CD [11]. A literature review published in 2015 summarized most studies that focused on the CD patient support [2]. The results of the Ludvigsson et al. review confirmed that the first information about CD and treatment with a GFD should be given at the time of diagnosis or shortly thereafter. In addition, patient information and an increased level of awareness may improve symptoms in patients with CD [2]. Similar results were obtained from a cross-sectional study to determine the effect of education on the knowledge of patients with CD in Iran by using questionnaire pre-/posteducational meetings [12]. They reported that educational meetings could increase the knowledge of CD patients as a treatment strategy and may lead to improvements in patients' health [13]. On the other hand, an Indian study pointed to the importance of implementing the labelling laws and guidelines for an acceptable amount of gluten content among the patients [12]. Two Swedish randomized clinical trials have shown that patient education may have an impact on both gastrointestinal symptoms and the general well-being of patients with CD [14, 15]. In addition, they concluded that briefly informing the patient about the function of the small intestine is likely to help patients understand their symptoms, as well as the need to adhere to a GFD [14, 15].

In Jordan, to date, few studies have been published on CD overall, as mentioned in the Introduction, and this is the first study concerning the role of the educational intervention on the KAP among parents of CD patients. This study assesses KAP among parents of CD children, is aimed at increasing the education level of the parents with regard to CD via educational sessions, and evaluates the effect of increasing awareness of parents' KAP and children adherence to GFD.

## 2. Materials and Methods

### 2.1. Study Design

**Figure 1:**
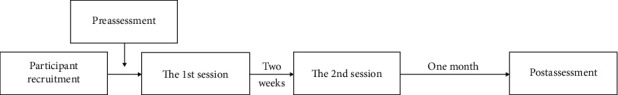
The study design of educational intervention for the parents of CD patients.

This study was a quasiexperimental, pre-/posttesting design which evaluated the effect of a four-week educational intervention on parents' KAP and children adherence to GFD. The test-retest method was used alongside within-group analysis. Two educational sessions were developed and conducted as part of a formal collaboration with the Celiac and Non-Celiac Care Providers Society (CCCPS) in Jordan. The patient's parents completed the baseline assessment before starting the educational session, then after two weeks, the parents attended the second session. Postassessment was completed after one month of the second educational session as shown in Figure 1.

### 2.2. Parent Recruitment and Study Population

Between November 2019 and January 2020, in collaboration with the CCCPS, registered patients were screened (*n* = 270). Invitation letters were sent to parents who met the inclusion criteria (*n* = 100), which were as follows: definitive diagnosis of CD for patients aged between 2 and 15 years old, by gastroenterologists through serologic tests (tTG and IgA) and small intestinal sampling (according to Marsh's classification) [16]. Inclusion criteria also included those who were willing to attend all the sessions and complete pre-/postassessment. Exclusion criteria included an unconfirmed CD diagnosis.

### 2.3. Settings

The education session was held as group sessions (20 parents in each group), at Haya Princess Centre, Amman, between February and March 2020.

### 2.4. The Educational Intervention

The contents of the educational sessions were based on recent up-to-date and global reliable training guidelines, Rothwell's training design principles [13], and the main guidelines for improving performance in the workplace [17]. The educational sessions were done in two different periods (3-4 hours each), followed by question and answer sessions, clinical counselling individually, and nutrition counselling for 30 minutes. The education material included the use of images, statements of goals, and questions related to each section. The presentation method was in accordance with the principles of instruction based on deductive, known to unknown, and generic to specific sorting methods. The content included clinical information related to CD history, definition, epidemiology, symptoms, and diagnosis. The nutritional part included information related to the GFD alternatives, allowed and not-allowed food, food additives, and meal planning training. Finally, gluten-free medications, gluten-free cosmetic information, and the approved recourses of information were also explained. Specially designed booklets were given to every participant including all the educational material in the Arabic language.

### 2.5. The Assessment Tools

The questionnaire has 31 questions in total, within four sections. The first section included seven questions regarding sociodemographic characteristics: child's age, gender, BMI, and disease duration. Additional information is the parent's education level, information source, and family income. The second part of the questionnaire was composed of 13 questions about the disease knowledge including definition, epidemiology, symptoms, diagnosis, and treatment (maximum score of 37); responses were scored as follows: correct response: 1, incorrect response: 0, and do not know: 0. Bias induced by guessing was reduced by including a “do not know” as an answer choice, while the questions that were based on the four-item Likert scale (strongly disagree, agree, and strongly agree) were scored from 0 to 3. Based on the score obtained, parental knowledge level was classified into three categories as poor (<60%), fair (60-75%), and good (>75%). The third section was composed of six questions related to the parent's attitudes and practices (maximum score of 17); it was classified into two categories as high and low risk (<50% and ≥50%, respectively). The final section was composed of five questions related to the child's adherence to the GFD (maximum score of 18), and it was classified into two categories: poor and good adherence (<50% and ≥50%, respectively). The questions were based on the 4-item Likert scale (no never, scarcely, sometimes, and always), which was scored from 0 to 3.

### 2.6. Reliability and Validity of the Questionnaire

The assessment tool used in this study was a questionnaire designed by the research team. Reliability was confirmed by Cronbach's alpha analysis using a test-retest method. The questionnaire was tested on a pilot sample of 20 patient's parents. Parents were contacted two weeks later and completed the questionnaire again (pre-/posttest reliability was performed). The questionnaire was reliable with regard to overall internal reliability (Cronbach′s alpha = 0.82) and test-retest reliability of 0.75. Gastroenterologist and nutritionist groups were consulted and asked to review the questionnaire, and they were able to comment and confirm validity. Content validity of the questionnaire was assured using the translation back-translation method. The questionnaire was translated from English to Arabic by a bilingual researcher (Arabic, English). To assure the exactitude of translation, another bilingual researcher retranslated the questionnaire back from Arabic to English. The two English versions (original, translated-back translated) were compared to assure that the meaning of the items in the two versions did not change. An expert panel examined the Arabic version of the overall questionnaire.

### 2.7. Statistical Analysis

The sample size calculation indicated that a sample of 40 participants would achieve a statistical power equal to 80% and a two-sided significance of 5% (i.e., two-sided *p* value less than 0.05) for detecting a difference of 0.3–0.4 between proportions (i.e., 30%-40% difference in KAP and/or adherence between pre intervention and post intervention) [18].

The data were analysed using IBM SPSS Statistics for Windows, Version 25.0 (IBM Corp., Armonk, NY, USA). Descriptive statistics, including frequencies, percentages, and crosstabulations, were obtained to measure the distribution of KAP and patient adherence according to the different social and demographic factors. Within-group differences and post intervention were detected by marginal homogeneity tests for three category variables and the McNemar test for two category variables. Mann–Whitney *U* was used to test the pre-/post intervention score. The significant level (*p* value) in this study is <0.05.

### 2.8. Ethical Approval

The study was approved by the Institutional Review Board and the ethical committee of Petra University, Amman, Jordan (Grant number: 4Q/1/2020).

## 3. Results

### 3.1. Participant Characteristics

**Figure 2:**
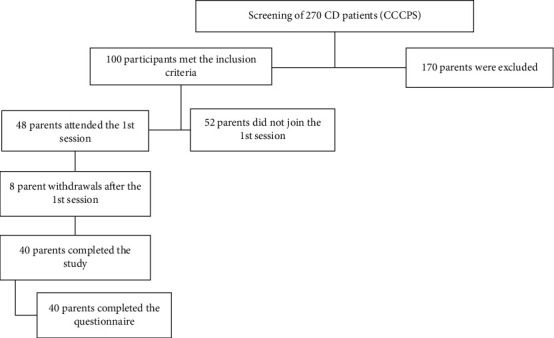
Flow diagram of CD parent participation.

A total of 270 CD cases of the registered family on CCCPS were screened (we searched for the inclusion criteria of the registered people on CCCPS). One hundred parents met the inclusion criteria of the study while 48 parents signed the consent form and attended the first session. Forty parents continued to the second session of CD; eight of them withdrew after the first session as shown in Figure 2. Parents who did not attend both sessions or did not fill in the questionnaire before or after the education sessions were excluded from the analysis.

The sociodemographic characteristics of CD patients and their parents indicated that the majority of the parents who participated in the study had children patients (57.5% boys and 42.5% girls) more than 10 years old. Regarding the CD patient's parents, 47.5% of the mothers completed high school or less and 70% of the fathers completed more than high school as shown in Table 1. The main source of the parent's information was either a gastroenterologist clinic (40%) or the internet/social media (40%), while the dietician had the lowest percent (20%).

### 3.2. Relationship between KAP Categories among CD Parents, and Patient's Adherence and the Sociodemographic Characteristics of CD Patients and Their Parents

According to the parents and patients' sociodemographic characteristic, the result indicated that there were no significant differences by nutrition knowledge categories in the sociodemographic characteristics of CD patients and their parents, except for information source (*p* value = 0.02). Parents who have poor or fair knowledge reported that they received their information from the internet or social media (55.6% and 44.4%, respectively), while those who have good knowledge obtained their information from the gastroenterologist clinic (66.7%) and were less likely to report using the internet or the social media as a source of information, as shown in Table 2. Similarly, there was no significant difference reported in the parents' attitudes and practices based on sociodemographic characteristics of CD patients and their parents, as shown in Table 3.  Table 4 shows that there was a significant difference in the patient's adherence to the GFD based on the duration of disease (*p* value = 0.05), with those with a longer duration of disease being likely to report better adherence.

### 3.3. The Difference in Parent's KAP and Patient's Adherence Pre-/Post Intervention

The results indicated that the median of all dimensions (KAP among CD parents and patient's adherence) increased significantly (*p* value ≤ 0.001, for all) after the intervention as shown in Table 5. In addition, as shown in Table 6, the categories of nutrition knowledge level were significantly different after the intervention (*p* value ≤ 0.001). Before the educational intervention, almost a quarter of the sample had poor knowledge. After the intervention, people were classified in the fair and excellent knowledge categories only, as well as the parent's attitudes and practices and the patient's adherence to GFD (*p* value was 0.001 and 0.001, respectively). After the intervention, all parents reported having low-risk attitudes and practices, which was significantly different from baseline. Moreover, the children moved to reporting good adherence after the intervention.

## 4. Discussion

### 4.1. Relationship between KAP Categories among CD Parents, and Patient's Adherence and the Sociodemographic Characteristics of CD Patients and Their Parents

This study was carried out to evaluate the CD patient's parents' KAP and the adherence of the CD patient's pre- and posteducational intervention regarding CD symptoms, diagnosis, treatment, GFD, and other gluten resources rather than food. In this study, 40 parents completed the study and attended both sessions. The rest of the parents could not attend and continue with the program due to a reported lack of time.

The results indicated that there was no significant relationship between the parent's knowledge regarding CD definition, symptoms, diagnosis, and treatment such as allowed food and not-allowed food and sociodemographic characteristics of the patients and their parent (*p* value > 0.05) except for the information source of the family (*p* value = 0.02). Our results indicated that more than half of parents who have poor nutrition knowledge receive their information from the internet; these results are in agreement with results reported by Tomlin et al. [19].

Unfortunately, parents frequently receive outdated, inaccurate, and/or conflicting information from internet resources (based on their response regarding the source of information) which confused and frustrated patients, who unnecessarily restrict certain foods, thus limiting the variety and nutritional quality of their diet. The qualified health care professionals, especially the dietician, have extensive academic and practical background regarding the role of food and nutrition in the prevention, treatment, and progression of acute and chronic diseases and how disease and treatment affect food and nutritional needs; food composition preparation information; socioeconomic, psychologic, and educational factors that affect food choices and nutrition behaviour of people across their lifespan; and counselling skills to translate scientific information into laymen's terms and assist clients in gaining knowledge, self-understanding, improved decision making, and behavioural changes. So they can provide comprehensive nutrition therapy for the patient [20].

However, some patients and their parents may not receive enough education sessions and health support from the health care professionals; this might make them to resort to unreliable, easy, and fast sources of information such as social media or unscientific web pages [19]. Our results agreed with US survey results of 253 adults: they reported that 71% found information about the GFD from books, support groups, family, friends, and the internet compared with 30% from physicians, and 66% of CD patients were referred to a dietitian, but a large sample (88%) claimed that no useful information came from the dietitian but came from the celiac support groups like societies [21]. Another study was in agreement: the internet is a major influential source of the knowledge level of parents who have patients suffering from CD [19].

Moreover, our results reported that there was no significant relationship between the parent's attitudes and practices and the sociodemographic characteristics of the CD patients and their parents, whereas the patient's adherence was associated significantly with the onset of disease only (*p* value = 0.05). A systematic review indicated that the effect of patient's age at CD diagnosis is less clear in many studies and not associated with patient adherence [22]. In line with our study, two cross-sectional studies have found that adherence improves with illness duration [23, 24]. Following GFD is challenging for many reasons such as wheat and wheat-based products being major components in Middle East meals especially Jordan and hectic lifestyles resulting in more meals being eaten outside the home even for school-aged children. Also, the hidden wheat, such as in seasonings, flavourings, modified food, and other products (medication and cosmetics), is unknown for most people, and for most patients, even their families are unaware of these facts. Finally, GFD cost is higher than other food in most countries including Jordan, and obtaining these special foods is difficult for some patients. Therefore, adopting a new lifestyle needs time and effort from the parents.

### 4.2. The Difference in KAP and Patient's Adherence Pre-/Post Intervention

The results indicated that the median of KAP among the parents and the patient's adherence increased significantly after the intervention. About one-third (37.5%) of participants had poor knowledge before the sessions, but after the sessions, no poor knowledge score was detected. This result may refer to the benefits of the small sample size that opens a good chance for all participants to understand the sessions and ask about more information individually after each session. The percentage of people who have good knowledge increased significantly from 22.5% to 87%. Our results agreed with Barzegar et al. study. They reported that the mean scores for treatment, epidemiology, and diagnosis before training for 90 CD patients were 7.16, 2.72, and 6.81, respectively, while the average after the training changed to 8.98, 7.16, and 9.06, respectively [13]. In addition, one study reported that increasing the knowledge level of professionals has a positive effect on the older age of CD patients and improves the outcome, and the health professionals are the ones mainly responsible of old age patients [25].

Few studies discussed the difference in knowledge for patients or their parent's pre-/posteducational intervention. However, similar to our findings, before education sessions which were reported by a cross-sectional study of 50 CD parents in Rajasthan reported an average knowledge score (less than 75%) for 86% CD parents [26]. Another study in the United States reported lack of awareness for CD patients at the symptom's onset time [27].

The percentage of parents with low-risk levels was increased after education sessions. Also, all patients had good adherence to GFD after the education sessions.

The patient's adherence results were considered as the logical outcomes for the change in parents' KAP after the education. Improved attitudes and practices of the patient's parents were considered as crucial social support that emphasized diet adherence and reduction of relapse [28].

Education is a very important part of any dietary change, not just for the health care personnel but also for the surrounding environment of CD patients (including nannies, school staff, and family). To the greatest extent possible, the patient and his/her family should be allowed to actively participate in decisions regarding the care of CD [2]. This awareness helps the patients to adapt to the new lifestyle better and faster, and it may improve symptoms in patients with CD [2]. Garg and Gupta reported that parents who have a higher degree of knowledge about CD, GFD adherence, and complications have more compliant CD patients [29]. In contrast, poor compliance with a GFD can have adverse health outcomes including ongoing symptoms and the development of complications.

## 5. Conclusion

This study found that increasing the knowledge level for CD patients' parents regarding all clinical and dietary aspects of CD could improve parents' attitudes and practices and consequently improve the patient's adherence to GFD. Baseline nutrition knowledge among the parents was associated with the information source, while the patient's adherence was associated with the duration of disease. This study suggested increasing the knowledge level of the CD patient's parents (for patients at a young age) through special education programs, conducted at the time of diagnosis. This study also suggests the potential for activation of the dietician role in nutrition counselling for CD patients besides the gastroenterologist role. Finally, the study suggests the need for an active role of CD societies in Jordan and to encourage them to cooperate with the education sectors to spread awareness and raise the knowledge level of CD patients and their families, because increasing CD knowledge among the parents and the patients may improve the symptoms and reduce the likelihood of complications in the long term, which will also have implications for health care costs.

### 5.1. Strength and Limitations

This study is the first study in Jordan aimed at providing educational intervention for CD patients.

Providing an educational program for parents of CD patients had a significant effect on increasing knowledge and awareness regarding CD symptoms, diagnosis, and treatment diet restrictions. This intervention also improved their patients' attitudes and practices and consequently affected children's adherence positively. Despite the strengths of this study, the small sample size is one of the limitations, and we also did not use a randomized design, with the inclusion of a control group. However, the core purpose was delivering an awareness education campaign to the parents. So the option of including a control group was considered ethically unsound. To scale up this intervention, more sessions would be needed in a range of different locations. In addition, this study included only parents of CD patients (children) and did not include medical staff (primary care professionals). Moreover, the adherence assessment was a self-reported assessment. Therefore, increasing the generality of findings including health professionals (primary care) regarding the nutritional knowledge, attitudes, and practices related to CD and performing similar studies with a larger sample size are recommended. Moreover, longitudinal studies are recommended examining the effect of education over a longer time and exploring whether CD patients and the parents need education sessions periodically.

## Figures and Tables

**Table 1:** Sociodemographic characteristics of CD patients and their parents.

Characteristics		*N* (%)
Child's gender	Boy	23 (57.5)
	Girl	17 (42.5)

Child's age	<5 years	7 (17.5)
	5-9 years	15 (37.5)
	≥10 years	18 (45.0)

Mother's education	High school or less	19 (47.5)
	More than high school	21 (52.5)

Father's education	High school or less	12 (30.0)
	More than high school	28 (70.05)

Monthly family income (JD)	<500	21 (52.5)
	500-700	14 (35.0)
	>700	5 (12.5)

Onset of disease	<1 year	13 (32.5)
	≥1 year	27 (67.5)

Information source	Gastroenterologist clinic	16 (40.0)
	Dietician	8 (20.0)
	Internet or social media	16 (40.0)

JD: Jordanian dinar ($1.71).

**Table 2:** Relationship between nutrition knowledge categories among CD parents and the sociodemographic characteristics of CD patients and their parents.

Characteristics		Poor knowledge *n* (%)	Fair knowledge *n* (%)	Good knowledge *n* (%)	*p* value
Child's gender	Boy	9 (39.1)	10 (43.5)	4 (17.4)	0.66
	Girl	6 (35.3)	6 (35.3)	5 (29.4)	

Child's age	<5 years	3 (42.9)	4 (57.1)	0 (0.0)	0.44
	5-9 years	6 (40.0)	4 (26.7)	5 (33.3)	
	≥10 years	6 (33.3)	8 (44.4)	4 (22.2)	

Mother's education	High school or less	5 (26.3)	8 (42.1)	6 (31.6)	0.28
	More than high school	10 (47.6)	8 (38.1)	3 (14.3)	

Father's education	High school or less	5 (41.7)	5 (41.7)	2 (16.7)	0.84
	More than high school	10 (35.7)	11 (39.3)	7 (25.0)	

Monthly family income (JD)	<500	6 (28.6)	10 (47.6)	5 (23.8)	0.28
	500-700	5 (35.7)	5 (35.7)	4 (28.6)	
	>700	4 (80.0)	1 (20.0)	0 (0.0)	

Onset of disease	<1 year	6 (46.2)	7 (53.8)	0 (0.0)	0.06
	≥1 year	9 (33.3)	9 (33.3)	9 (33.3)	

Information source	Gastroenterologist clinic	4 (26.7)	1 (6.7)	10 (66.7)	0.02
	Dietitian	7 (43.8)	3 (18.8)	6 (37.5)	
	Internet or social media	5 (55.6)	4 (44.4)	0 (0.0)	

JD: Jordanian dinar ($1.71). Poor knowledge < 60; fair knowledge 60-75; good knowledge > 75.

**Table 3:** Relationship between attitude and practice categories among CD parents and the sociodemographic characteristics of CD patients and their parents.

Characteristics		Low risk *n* (%)	High risk *n* (%)	*p* value
Child's gender	Boy	4 (17.4)	19 (82.6)	0.98
	Girl	3 (17.6)	14 (82.4)	

Child's age	<5 years	2 (28.6)	5 (71.4)	0.56
	5-9 years	3 (20.0)	12 (80.0)	
	≥10 years	2 (11.1)	16 (88.9)	

Mother's education	High school or less	4 (21.1)	15 (78.9)	0.57
	More than high school	3 (14.3)	18 (85.7)	

Father's education	High school or less	4 (33.3)	8 (66.73)	0.08
	More than high school	3 (10.7)	25 (89.3)	

Monthly family income (JD)	<500	5 (23.8)	16 (76.2)	0.44
	500-700	1 (7.1)	13 (92.9)	
	>700	1 (20.0)	4 (80.0)	

Onset of disease	<1 year	2 (15.4)	11 (84.6)	0.81
	≥1 year	5 (18.5)	22 (81.5)	

Information source	Gastroenterologist clinic	3 (42.9)	1 (14.3)	0.92
	Dietitian	13 (39.4)	7 (21.2)	
	Internet or social media	16 (40.0)	8 (20.0)	

JD: Jordanian dinar ($1.71). High risk < 50; low risk ≥ 50.

**Table 4:** Relationship between patient's adherence categories and the sociodemographic characteristics of CD patients and their parents.

Characteristics		Poor adherence *n* (%)	Good adherence *n* (%)	*p* value
Child's gender	Boy	14 (60.9)	9 (39.1)	0.90
	Girl	10 (58.8)	7 (41.2)	

Child's age	<5 years	2 (28.6)	5 (71.4)	0.06
	5-9 years	12 (80.0)	3 (20.0)	
	≥10 years	10 (55.6)	8 (44.4)	

Mother's education	High school or less	10 (52.6)	9 (47.4)	0.37
	More than high school	14 (66.7)	7 (33.3)	

Father's education	High school or less	7 (58.33)	5 (41.7)	0.89
	More than high school	17 (60.7)	11 (39.3)	

Monthly family income (JD)	<500	13 (61.9)	8 (38.1)	0.49
	500-700	7 (50.0)	7 (50.0)	
	>700	4 (80.0)	1 (20.0)	

Onset of disease	<1 year	8 (61.5)	5 (38.5)	0.05
	≥1 year	11 (40.7)	16 (59.3)	

Information source	Gastroenterologist clinic	7 (29.2)	5 (20.0)	0.19
	Dietitian	9 (65.3)	3 (18.8)	
	Internet or social media	16 (40.0)	8 (20.0)	

JD: Jordanian dinar ($1.71). Poor adherence < 50; good adherence ≥ 50.

**Table 5:** Parent's KAP and patient's adherence pre- and post intervention (*n* = 40).

		Minimum	Maximum	Median
Pre	Parent's nutrition knowledge	45.95	89.19	64.86
	Parent's attitudes and practices	5.88	100.00	70.59
	Children's GFD adherence	5.56	100.00	41.67

Post	Parent's nutrition knowledge	62.16	91.89	91.89
	Parent's attitudes and practices	70.59	100.00	100.00
	Children's GFD adherence	55.56	100.00	77.78

*p* value is ≤0.001 (pre-/post intervention).

**Table 6:** Frequency of pre-/post intervention for the different categories of parent's knowledge, attitudes, and practices and children's adherence to GFD.

	Pre intervention *n* (%)	Post intervention *n* (%)	*p* value
Parent's nutrition knowledge			≤0.001
Poor knowledge < 60	15 (37.5)	0	
Fair knowledge 60-75	16 (40.0)	13 (13)	
Good knowledge > 75	9 (22.5)	78 (87)	

Parent's attitudes and practices			0.014
High risk < 50	7 (17.5)	0	
Low risk ≥ 50	33 (82.5)	40 (100)	

Children's adherence to GFD			≤0.001
Poor adherence < 50	24 (60.0)	0	
Good adherence ≥ 50	16 (40.0)	40 (100)	

## Data Availability

The data used to support the findings of this study are available from the corresponding author upon request.

## Abbreviations

CCCPS: Celiac and Non-Celiac Care Providers Society
CD: Celiac disease
CI: Confidence interval
GFD: Gluten-free diet
KAP: Knowledge, attitudes, and practices.
